# Effectiveness of platelet-rich fibrin (PRF) block graft in managing intra-bony defects: A case report

**DOI:** 10.6026/973206300211817

**Published:** 2025-07-31

**Authors:** Surthi Senthi, Harini S, Saranya Mohan, Vijayalakshmi Rajaram, C. Burnice Nalinakumari, D. Shunmuga Prasanth, Jaideep Mahendra

**Affiliations:** 1 Department of Periodontics, Meenakshi Ammal Dental College and Hospital, Chennai, Tamil Nadu, India

**Keywords:** Intra-bony defects, platelet-rich fibrin (PRF), bone fill

## Abstract

Periodontal disease is a multifactorial condition affecting tissues and supporting bone, potentially leading to tooth loss. Therefore, it is of interest to remove inflammatory tissues and restore health, utilizing techniques like guided tissue regeneration, bone grafts, and root surface bio-modifications. Platelet-rich fibrin (PRF) is a promising biomaterial due to its autologous nature and regenerative potential. This case report evaluates PRF block grafts in managing intra-bony defects, assessing pocket depth reduction, attachment level gain, and radiographic bone fill. The results showed significant clinical and radiographic improvements suggest PRF block grafts as an effective modality for periodontal regeneration. Data shows PRF as a viable alternative to conventional grafting materials. PRF may enhance healing and improve periodontal prognosis in treating intrabony defects.

## Background:

Periodontal disease is considered as a multifactorial disease that destroys the attached tissue and supporting bones which may lead to tooth loss. The aim of periodontal therapy is to remove the destroyed inflamed tissues and regain healthy periodontal tissue [1]. Periodontal tissue regeneration can be accomplished in a variety of ways, including guided tissue regeneration, bone grafts, and root surface biomodifications. Regaining all of the periodontal tissues-fibroblasts, osteoblasts, periodontal ligament fibers, and cementum is necessary for periodontal regeneration. Platelets and cytokines have been observed to collect within the fibrin clot during wound healing, and the platelets release a number of growth factors into the surrounding tissues. Wound healing is greatly aided by the growth factors and cytokines that are present in platelets. Fibrin, vitronectin, and fibronectin are secreted by platelets and serve as a matrix for connective tissue. These all suggest that using platelet concentrates can be quite effective in promoting the regeneration of periodontal tissue [2]. Platelet Rich Fibrin (PRF) has been used in various fields of dentistry. It is used to deliver growth factors directly to the tissue surface. It can be used in topical and injectable forms. Platelet concentrates are obtained by centrifuging blood samples to separate platelet from plasma. There are various generations of platelet concentrates whereas PRF is a second generation platelet concentrate. Other various types of PRF are Advanced PRF (A-PRF), Advanced PRF + (A-PRF+), Injectable PRF (i-PRF), Titanium PRF (T-PRF) and Growth Factor Impregnated PRF [3]. The use of second-generation platelet concentrate, leucocyte and platelet rich fibrin (L-PRF) to create a graft with high concentration of growth factors, platelets and leucocytes may enhance the development of mature lamellar bone. The L-PRF block is prepared by mixing a particulated biomaterial with chopped L-PRF membranes at a 50:50 ratio and adding i-PRF to bind the components together [4]. In this case we used PRF block, which is a combination of PRF gel and bone graft. In this study, we use PRF block which is made up of 10ml PRF gel combined with de-mineralized freeze-dried bone allograft.

## Case report:

A 42-year-old female patient came to the Department of Periodontology with a complaint of generalized bleeding gums while brushing. The patient was systemically healthy. Upon clinical examination, there was a pocket depth (PD) of 8mm and Clinical Attachment Loss (CAL) of about 8mm in Root Canal Treated 46. Radiographic findings reveal angular bone loss in the mesial aspect of 46 with widening of the periodontal ligament (PDL) space.

## PRF preparation:

10 ml of venous blood was collected from the patient and prepared without adding any anticoagulants and then this specimen underwent immediate centrifugation at a speed of 2500 rpm for 12 minutes. During the centrifugation process, when the blood comes into contact with the test tube wall, the platelet gets activated, initiating the coagulation cascade. After centrifugation, the product consists of three layers: The top layer is platelet poor plasma layer, the middle layer is the PRF clot, while the bottom layer is red blood cells (RBCs). The fibrin clot obtained after centrifugation is discarded from the tube and the attached RBCs were detached from it and removed [3]. This PRF clot was mixed with dFDBA to form the PRF block.

## Surgical procedure:

After giving local anesthesia, crevicular incisions were given using no 15 bard-parker blade. Flaps were elevated using molt no.15 periosteal elevator. The exposed root surface was thoroughly debrided and root planed dFDBA (Osseograft) was emptied into a dappen dish and autologus PRF was prepared and mixed with Osseograft and a bone block was made and was condensed in the defect. The flap was then closed with 4'0' black braided silk sutures. A tension free primary closure was obtained. Periodontal pack was placed.

## Post-operative instructions:

Antibiotics and analgesics were prescribed for 5 days. Post-operative instructions were given. Patients were advised to avoid chewing in the area for 2 weeks and not to brush for 10 days. Suture removal was done after 10 days. Recall appointments were scheduled at 3 and 6 months after the surgery for soft tissue evaluation, and clinical variables. Patient was recalled after 6 months and radiographs were taken for evaluating the bone fill.

## Discussion:

Platelet rich fibrin (PRF) has been introduced by Choukroun *et al.* in 2001 [5] which belongs to a second-generation platelet concentrate. The PRF concentrates almost all the growth factors and platelets. The novel technique described in this study combines properties of bone blocks and particulated grafts reducing disadvantages of both. Combination of liquid fibrinogen along with PRF increases ease in handling and predictability of augmentation procedure. PRF gel can be mixed with bone graft together to form a biological matrix which promotes the migration of osteoprogenitor cells to the center of graft and induces neo-angiogenesis [5]. The PRF in the block is a matrix rich in activated platelets secreting wide range of bioactive molecules and growth factors including Bone Morphogenic Protein (BMP), Platelet Derived Growth Factor (PDGF), Insulin Growth Factor (IGF), Vascular Endothelial Growth Factor (VEGF), Transforming growth factor (TGF), Epidermal Growth Factor (EGF), Basic Fibroblast Growth Factor (bFGF), Transforming Growth Factor β1 (TGF-β1), activin-A, Bone Morphogenic Protein 4 (BMP-4), Hepatocyte Growth Factor (HGF), β Nerve Growth Factor (βNGF), and retinoic acid. These factors help in bone healing and regeneration [6].

They help in stimulating the in-vitro proliferation and differentiation of human oral bone mesenchymal stem cells in a dose dependent way but also induces mesenchymal stem cell migration as a response to the factors released. PRF shows beneficial properties of neovascularization [7]. This leads to faster maturation of augmented areas and reduced amount of biologically inactive scaffold. The scaffold and matrix were filled with liquid fibrinogen. This starts the coagulation cascade when in contact with PRF membrane. This process took place in less than 5 minutes and trapped the biomaterial into a retaining block. The block has a proof consistency, with light elasticity to adapt it to recipient site [8]. Sculean *et al.* [9] in the systematic review, described that most studies have demonstrated superior histologic healing following the combination of barrier membranes and grafting materials than following open flap debridement; hence in this case with the use of sticky bone, GTR membrane was also placed for better outcome. In this study the mean volumetric resorption rate of the graft was 15.6%. The limited resorption indicates the integrity of block was maintained during integration process. The bone graft and PRF block are made from a mixture of particulated graft material with autogenous bone or with PRF and liquid fibrinogen, respectively. The main advantage is, for PRF block no autologous bone had to be harvested from second site. The composition of block was dFDBA graft and PRF gel seemed to be beneficial to cover the augmented site with PRF to protect the collagen membrane and prevent the contamination of the graft, as it acts as a barrier and in time integrates with surrounding soft tissues.

## Conclusion:

Platelet-rich fibrin (PRF) and demineralized freeze-dried bone allograft (dFDBA) demonstrated superior outcomes over conventional procedures. The combined PRF approach effectively treated endo-perio lesions, showing promising bone augmentation results. PRF block appears to be a successful, safe, and predictable protocol with high feasibility and low morbidity. Further investigation and clinical research are needed to validate its long-term effectiveness. This combined therapy may offer a reliable alternative for periodontal regeneration.

## Sources of support:

Nil
